# "We are still tired": staff and administrators’ experiences during the COVID-19 pandemic within California residential care facilities for older adults

**DOI:** 10.1186/s12877-023-04537-2

**Published:** 2023-12-18

**Authors:** Kaitlin O. Bahr, Grishma P. Bhavsar, David Zhao

**Affiliations:** 1 Public Health Program, California State University Northridge, 18111 Nordhoff St., Northridge, CA 91330 USA; 2 Health Administration Program, California State University Northridge, 18111 Nordhoff St., Northridge, CA 91330 USA

**Keywords:** Residential care facilities for older adults, COVID-19, Pandemic, Emergency preparedness, Staff, Morale, Stress, Long-term care, Older adults

## Abstract

**Background:**

Extensive research has been conducted on the impacts of the COVID-19 pandemic on long-term care workers in specialized care facilities. However, little is known about the impacts faced by facilities that provide generalized long-term support and care, such as residential care facilities for older adults (RCFs). This study describes the challenges experienced by staff and administrators of RCFs during the COVID-19 pandemic.

**Methods:**

An electronic questionnaire collecting data using both closed- and open-ended questions on staff experiences was sent to 5,721 unique RCF administrator emails within the state of California between June-December 2021. Email addresses were obtained from the public database of RCFs available through the California Health and Human Services Open Data Portal. Descriptive statistics were calculated on quantitative data regarding staff preparedness training, access to resources, and administrators’ confidence in meeting recommended guidelines during the pandemic. Inductive thematic analysis was conducted on qualitative data regarding the confidence levels in meeting pandemic guidelines and challenges faced related to staff stress and morale.

**Results:**

A total of 150 RCF administrators across California (response rate of 2.6%) completed the survey. Over three-fourths of respondents indicated their facilities had a designated staff member to train other staff members on emergency preparedness plans and the most frequently used resources during the COVID-19 pandemic were the Department of Social Services Community Care Licensing Division (88.7%), the county health department (86.7%), and the Centers for Disease Control and Prevention (80.7%). Administrators felt least confident in their facilities’ ability to maintain adequate staffing (52.0%), communication with nearby hospitals (62.1%) and communication with state and local public health officials (69.8%) during the pandemic. Three central themes emerged from the thematic analysis on staff stress and morale: (1) physical safety, mental and emotional impact of the COVID-19 pandemic; (2) staffing issues; and (3) challenges with guidelines in managing the ongoing pandemic.

**Conclusions:**

Findings from this research study can be used to actively target training resources for facility administrators and staff that have been identified as most frequently used and relevant for emergency preparedness in these understudied facilities. Additionally, developing a better understanding of the staffing stress and morale difficulties in RCFs can provide insight on how policymakers can assist these critical facilities in better preparing for future crises.

## Background

Since the beginning of 2020, the SARS-CoV-2 (COVID-19) pandemic has heavily impacted the lives of those who work and live in long term care facilities. Vulnerable populations of older adults living in shared spaces, many of whom have comorbid conditions, require frequent person-to-person contact for the delivery of personal care [1]. These factors contributed to frequent COVID-19 outbreaks in long term care facilities, high levels of mortality among residents, and high levels of infection among long term care staff [2–8]. Many studies have found that COVID-19 morbidity and mortality were exceptionally high in long term care facilities, accounting for a disproportionate number of cases and deaths when compared with the total population [5–11].

Studies have also shown a significant effect of the pandemic on the mental health of residents in skilled nursing homes and larger assisted living facilities [1, 3, 12–18]. Fearful of infection, living in isolation and unable to see their loved ones, residents and their families were found to experience negative psychological impacts, such as loneliness, post-traumatic stress, anxiety, and depression [2, 13–15, 19, 20].

The psychological and emotional well-being of staff in these facilities were also substantially impacted during the pandemic [1, 3, 16]. Staff were faced with increased workload, adopting additional responsibilities, mental and physical exhaustion and high burnout, in addition to the increased risk of contracting COVID-19 [1, 2, 13, 16, 21–23]. Administrators in skilled nursing homes had to manage chronic staff shortages, high levels of turnover, information overload, and communication gaps with outside agencies regarding constantly changing guidelines [1, 2, 13, 21, 22].

Prior research has predominantly examined the impact of the COVID-19 pandemic on the workforce of long-term care facilities that provide complex medical care, such as skilled nursing facilities. Fewer studies, however, have focused on the impact on residents and staff at facilities that provide generalized day-to-day support and care, such as residential care facilities for older adults (RCFs), despite the large population they serve.

RCFs for older adults, known in California as residential care facilities for the elderly or RCFEs, represent a subset of long-term care options for older adults and provide non-medical care and supervision in a residential setting for those who require assistance, but do not require 24-h nursing care [24]. In California (CA) alone, there are over 7,400 licensed RCFEs which can provide housing and care for more than 185,000 residents [24, 25]. These facilities can range from small communities serving under 16 residents (representing 83% of RCFEs in CA), many of which are set in residential home-like settings, to larger facilities serving more than 100 residents [26]. As the number of California residents aged 85 and older is expected to at least double by 2050, the need for these facilities to provide quality care to residents will continue to grow in the coming years [24].

RCFs are faced with different licensing requirements and regulations than skilled nursing facilities and have been found in previous studies to have fewer resources to dedicate towards emergency preparedness, such as pandemic planning, than skilled nursing facilities [27, 28]. This limitation, coupled with the lack of comprehensive studies on the impact of COVID-19 on administrators and staff in RCFs, represent important gaps in the literature focused on care for aging populations. It is important to assess the impact of COVID-19 on the workforce of RCFs to identify current deficiencies and future needs of these facilities, and adequately prepare for future emergency situations such as another pandemic. The aim of this study focused on assessing perceived challenges experienced during the pandemic by staff and administrators from the point of view of RCF administrators. Better understanding of the challenges experienced by this unique population will help to improve support systems and practices for these important facilities in the years to come.

## Methods

Data for this study was collected using an electronic survey between June and December 2021. Participants were recruited from the database of RCFs (designated as RCFEs in California) which is publicly available through the California Health and Human Services (CalHHS) Open Data Portal [25]. After removing duplicate and invalid email addresses from those listed in the database, a link to an anonymized self-completed electronic questionnaire was sent to 5,721 unique RCF administrator emails, from currently licensed facilities within the state of California. Individuals were invited to complete the electronic questionnaire using Qualtrics software if they were at least 18 years of age and currently serving as an administrator of a licensed RCF facility in California [29]. Reminder emails were sent to all those invited to participate in the survey, both 2 weeks and 3 weeks after the initial email.

The questionnaire included questions such as facility size, staff preparedness training, access to resources and staff experiences during the COVID-19 pandemic. Staff training questions included “Who trains staff members on the emergency preparedness plan?” [Designated member of staff at facility, Purchased resources from Department of Social Services (DSS) approved vendor, Other] and “How often is training provided on emergency preparedness to staff?” [Monthly, Quarterly, Annually, Other]. Access to resource questions asked participants to indicate what resource(s) they utilized to obtain current information about COVID-19 and what support/training resources they would find beneficial. Finally, staff experience questions included administrators’ perceived confidence on their facility’s ability to meet the recommended guidelines during the pandemic restrictions, as well as questions designed to collect qualitative data on staff experiences asking administrators to “provide any additional comments on your facility’s ability to meet the following recommended guidelines during the pandemic restrictions.” and “What, if any, challenges did your facility face during the COVID-19 pandemic related to staff stress and morale?”.

Descriptive statistics, including percentages and frequencies, were calculated with quantitative data on staff training, access to resources, and administrators’ perceptions of their facility’s confidence in meeting guidelines during the pandemic. Inductive thematic analysis, identifying themes directed by an open exploration of the data without a preexisting coding frame, was used with qualitative data on reported challenges to staff stress and morale and expanded questions on confidence in meeting pandemic guidelines [30]. Each of the three researchers identified codes based on responses and later debriefed, discussing and compiling codes into agreed upon overall themes, in a similar manner to prior studies on care home staff experiences during the pandemic [16].

## Results

A total of 150 responses were collected from RCF administrators across California between June and December 2021, representing a response rate of 2.6%. The majority of respondents were from smaller facilities serving less than 7 residents (70.7%, *n* = 106), 19.3% (*n* = 29) of respondents were from facilities serving 7–99 residents and 9.3% (*n* = 14) of respondents were from larger facilities serving 100 or more residents.

### Staff training and access to resources

When asked which options were utilized for staff member preparedness training, 114 respondents (76.0%) indicated that a designated member of staff at the facility trained staff members on the emergency preparedness plan, 17 respondents (11.3%) noted that resources for staff training on the emergency plan were purchased from DSS approved vendor, and 12 respondents (8%) reported that either a combination of these was used or the administrator was responsible for training. The largest proportion of respondents reported that emergency preparedness training was provided to staff quarterly (50%, *n* = 75), followed by annually (29.3%, *n* = 44) and monthly (16.7%, *n* = 25).

The most frequently reported resources utilized by administrators to obtain current information about COVID-19 were the Community Care Licensing (CCL) Division (88.7%), County Health Department (86.7%), Centers for Disease Control and Prevention (CDC) (80.7%), Department of Social Services (DSS) (79.3%) and the California Department of Public Health (62.0%). All other resources noted on the questionnaire (including California Assisted Living Association (CALA), California Provider Helpline, The “WIRE” – DSS CCL electronic newsletter) were utilized by less than a third of respondents.

When asked what support or training administrators would find beneficial, emergency preparedness webinars and workshops (58.7%); establishing communication lines with nearby hospitals, state and local public health officials (54.0%); conducting emergency preparedness exercises (52.0%); and mutual aid agreements with local/state/federal organizations (50.7%) were the most common responses.

### Confidence in meeting pandemic guidelines

Administrators felt most confident following guidelines related to maintenance of facilities (89.3%), food and water safety (89.3%) and communication with family (87.9%), and felt less confident in adequate staffing (52.0%), communication with nearby hospitals (62.1%) and communication with state and local public health officials (69.8%).

Of the 150 total respondents, 46 provided qualitative responses when asked for additional comments on their facility’s ability to meet recommended guidelines. Of these, 17.4% (*n* = 8) noted communication challenges or lack of support from the state/local/licensing agencies during the pandemic, such as “unclear, miscommunicated and overlapping instruction from Dept of Social Service” and that “The state issues guidelines as to what we must do, but provides no help in getting it done”. One respondent noted:

*“There was inadequate direct communication from State agencies to facilities during those first few months of the pandemic, then severely delayed guidelines followed. In speaking with other administrators throughout 2020, there was an overwhelming sense of being on our own to interpret and apply information from reliable sources, like the CDC, while trying to counteract the endless stream of misinformation.”*

Several respondents also reported staffing issues (10.9%, *n* = 5) and supply shortages (10.9%, *n* = 5), such as “...difficulty with keeping staff requires constant retraining” and “the thing that was lacking for our plan was weathering long term supply chain issues.” One respondent specifically noted challenges obtaining PPE, commenting that “…we have no special access to supplies other than the common citizen.” Another respondent also mentioned challenges with obtaining PPE:

*“Initially in March 2020 the Ombudsman and RCFE Community Care Licensing did NOTHING to help guide us to obtain supplies during shortages to meet PPE, sanitizing, and paper products. Licensing sent out weekly and monthly surveys to determine shortages, but essentially offered NO guidance on how to obtain supplies. Price gouging occurred on what little supplies were found by going to multiple sources. We did not feel supported.”*

Despite challenges, a portion of respondents also mentioned positive experiences with state/local/licensing agencies (13.0%, *n* = 6), such as the CDC and CCL being “very supportive” during an outbreak and the benefits of DSS-CCL in assisting RCFs to obtain “*…*PPE supplies especially at peak of the pandemic and also training and guidance.” One respondent pointed out the value of frequent DHS phone visits via Zoom, noting that “communication is key.” Another respondent mentioned that the county personnel and licensing department helped in letting them know “where to go and get what we need.”

### Challenges to staff stress and morale

Qualitative data on reported challenges to staff stress and morale were organized into a variety of subthemes fitting within 3 central themes: (1) physical safety, mental and emotional impact of the COVID-19 pandemic; (2) staffing issues; and (3) challenges with guidelines in managing the ongoing pandemic. Central themes and subthemes are described in Table 1, including the frequency and percentages of respondents noting each subtheme. For each central theme, a sample of relevant quotes from respondents is included below.

**Table 1:** Central themes and subthemes identified using inductive thematic analysis

**Central Theme** **(Frequency; % of respondents noting each central theme)**	**Subtheme**	**Frequency (%) of respondents noting each subtheme**^a^ **(*n* = 113)**
Physical safety, mental and emotional impact of the COVID-19 pandemic (*n* = 59; 52.2%)	Low morale/high stress/burnout	29 (25.7%)
	Staff safety/Fear of COVID-19	28 (24.8%)
	Isolation	9 (8.0%)
	Burden of responsibility for residents happiness/safety	7 (6.2%)
	Fear of infecting others (e.g., family, other staff, residents)	4 (3.5%)
	Loss/death of staff/residents	3 (2.7%)
Staffing issues (*n* = 30; 26.5%)	Staff shortages	27 (23.9%)
	Overworked staff	5 (4.4%)
Challenges with guidelines in managing the ongoing pandemic (*n* = 15; 13.3%)	Burden of required testing/vaccination/masking/safety guidelines	10 (8.8%)
	Confusing/conflicting/changing guidelines	3 (2.7%)

^a^Individual respondent comments that included more than one subtheme were only counted once in the central theme, but separately in the appropriate subthemes

The most commonly mentioned subthemes included “low morale/high stress/burnout”, “staff safety concerns/fear of COVID” and “staff shortages” (with 25.7%, 24.8% and 23.9% of respondents noting these, respectively).

#### Physical safety, mental and emotional impact of the COVID-19 pandemic

Many administrators noted staff safety concerns and fear of COVID-19 as one of the top challenges experienced to staff stress/morale during the pandemic. Comments included staff fears of “…each other, especially depending on who they spent their off time with.” And of “…caring for COVID positive or COVID exposed residents.” One respondent remarked:

*[My workers thought it was a death sentence to work with COVID patients. Fear was constant...It was a tougher battle to convince workers to work than fighting the virus.]*

In addition to staff fear for their own health, several administrators also noted general feelings of low morale, high stress and burnout of their staff during such a challenging time. Issues of “depression, fatigue” were mentioned by respondents with one commenting “lots of team discussions were needed to support and keep up morale.” One respondent remarked that many staff “were courageous and others also terrified and needed reassigned or time off.” Another spoke to the continued mental health burden noting, “It was hard- we are still tired.”

Respondents also noted other concerns related to physical safety, mental and emotional impact of the pandemic, including isolation, fear of infecting others, the overall burden of responsibility for resident safety and happiness and the loss or death of staff and residents. One respondent called out the “Immense pressure to keep people engaged and active mentally, physically and socially” and another mentioned the effects of “too much isolation (from family members and friends’ visitation) fear, depression, loneliness, quality of life decline”. Another respondent commented:

*[The heavy burden of ensuring safety of the community while also being concerned for family was stressful. The isolation and canceling of all events negatively affected morale for everyone.]*

#### Staffing issues

One of the most frequent challenges mentioned by respondents was related to staffing. Staffing concerns ranged from staff quitting or refusing to work, overworked staff, needing additional staff capacity to manage absences related to the pandemic, and staff shortages due to difficulties finding qualified/trained individuals to fill positions when staff quit. Respondents specifically noted “Staff outages due to symptoms, quarantine, test results taking up to 12 days to get back.” And employees walking off the job, as “they thought they might die.” One respondent mentioned, “There were hardly relief workers available so if a staff member were to get sick, it would be a great burden on the remaining staff. No one wants to be in a situation where they can’t leave to take care of their own family.” Another noted that during an outbreak that caused a facility lockdown “…it was two caregivers and myself working all shifts. We felt the labor shortage pretty hard.”

#### Challenges with guidelines in managing the ongoing pandemic

Lastly, among the issues noted, several respondents mentioned factors relating to the challenges with guidelines including the burden of required testing, vaccination, masking and safety guidelines; and confusing, conflicting and changing guidelines. Respondents reported it was “very stressful with conflicting guidelines to the public” and that the “constant changing of guidelines was difficult.” One noted that it took a while for staff to get vaccinated, as “some staff were scared and misinformed about the vaccine at the beginning.” Another respondent mentioned the difficulty staff had in wearing masks during long shifts and that staff “stressed to continue getting tested…even though many of them have transportation difficulties.” One administrator remarked on the challenges with staff compliance in following guidelines at first, as it was “very difficult to manage and try to mitigate danger around COVID when we have a set of rules/requirements we have to follow when the public goes by what I feel like is a less restrictive guideline than facilities.”

### Factors that helped alleviate stress/improve morale

Within the responses to challenges to staff stress and morale, respondents also mentioned positive comments on what helped staff stress/morale during the pandemic. Strategies that respondents’ felt alleviated stress and improved morale included: mutual support (*n* = 6), staffing model changes (*n* = 4), good communication (*n* = 3), and education/training (*n* = 2). Respondents noted that morale was kept up with “ongoing positive feedback”. Comments on staffing model changes included switching “to a live-in staffing model to reduce potential exposure.” One respondent remarked that “with increased education we noticed a significant improvement with staff stress and morale. The more confident staff became the overall morale increased.” Training and “rich communication” with the local health department, CDPH and DSS was also mentioned to have a positive effect on stress levels. One administrator commented:

*[We had informal meetings with employees, both singular and as groups, as often as possible. We talked not just about what we needed to do at our facility but our situations at home with our families and loved ones. We made sure to stress the importance of listening to direct sources of credible information, like the CDC, as opposed to cable news. Maintaining and boosting morale with staff, residents, and families is a constant hands-on affair, and easier than trying to recover it.]*

## Discussion

The findings from this study provide important insight into an understudied population of RCF administrators and staff regarding their training/resource needs in emergency preparedness and their experiences during the COVID-19 pandemic.

The majority of respondents reported that a designated member of the staff at the facility trained staff members on the emergency preparedness plan. This indicates that these designated staff members need to be identified and receive up-to-date communication and guidance on necessary components of an emergency preparedness plan and appropriate trainings based on licensing requirements. If the RCF staff members providing training and conducting emergency preparedness drills are not equipped with the tools and education needed to adequately inform their staff, staff can be ill-prepared in the event of an emergency. The overwhelming majority of respondents reported that staff are trained in emergency preparedness at least annually and many on a more frequent quarterly or monthly basis, which meets DSS CCL licensing requirements and helps to ensure staff are actively considering the facilities emergency preparedness needs [31].

It is essential that information on requirements and resources for support applicable to RCFs in a situation such as a pandemic/outbreak or other emergency are able to reach the administrators of these facilities. Relevant information on emergency situations specific to RCFs needs to be provided in resources widely referenced by RCF administrators, such as CCL, CDC, DSS, county health department and CDPH. Administrators of these facilities need to be trained on where relevant information and details for communications within each of these agencies is located.

When describing confidence levels in meeting pandemic guidelines, it is encouraging that the majority of respondents felt confident in the maintenance of facilities and food and water safety. This may be attributed to these factors being principal components in the licensing process of an RCF facility and essential pieces in maintaining licensing both during and outside of the pandemic period [31]. While the current study did not ask participants detailed information on how facilities were maintained or what exercises were conducted in the areas of food and water safety, ongoing training is useful in these areas to ensure compliance with licensing requirements.

Two areas participants felt least confident in were communication with local and state public health officials and communication with nearby hospitals. Communication issues with outside organizations such as licensing agencies (e.g., CCL/DSS) and public health/governmental agencies (e.g., CDPH, state) were also noted in the qualitative comments. This is consistent with previous studies highlighting a lack of clarity on communication of guidelines/requirements and support available during the pandemic to assisted living facilities/nursing homes [32]. Frequently changing guidelines from health officials has also been shown to contribute to reduced compliance due to confusion, exhaustion and frustration [33]. This represents a critical deficiency and an important opportunity for improvement. Guidelines for facilities must be relayed clearly, consistently, and through appropriate means to allow for timely access for facility administrators to manage their facilities and care for their residents. RCF staff and administrators also need to be familiar with where, how and who to contact to get support and aid from local, state and federal organizations or to obtain information relevant to their facilities. Increased outreach and better established mutual aid agreements by state and agencies that account for the unique nature of particularly smaller facilities can help to minimize these communication deficiencies and allow for these facilities to obtain better support. Training and collaboration with outside agencies such as hospitals has been shown to be beneficial at increasing interagency lines of communication, so future incorporations of these collaborative training opportunities with RCFs could be useful in filling these gaps as well [34].

Staff members’ reported safety concerns and the mental/emotional impact of the pandemic including fear of contracting COVID-19 and caring for COVID-positive patients are consistent with previous studies in various sectors of the care industry, including nursing homes, assisted living facilities, hospitals and likely exacerbated as many were not able to acquire adequate PPE and/or sanitation supplies in a timely manner, resulting in elevated stress levels and lowered morale and confidence [2, 3, 13, 16–23, 35]. In addition to physical concerns of staff, the mental load of isolation, burnout, burden of responsibility for residents and overall low morale experienced is consistent with previous studies and had a significant and lasting impact in care worker populations [2, 3, 13, 16, 21–23, 35]. Qualitative comments illustrate the potential continued effects on mental health experienced by this population. The high physical demands and emotional stress of direct-care workers has been documented well before the pandemic, and COVID-19 both exacerbated and helped to shine a light on the challenges experienced by this population [36]. Not only are future changes needed to better plan, prepare and build capacity for mental health support for care workers, but those that have been working throughout the last several years may currently need expanded services to deal with the fatigue, burnout and devastating toll on mental health to which the pandemic contributed.

Staffing issues in the direct-care workforce, including staff at RCFs, have been recognized for some time [36]. This population of workers have difficult jobs, that are frequently low paid, include high levels of physical and emotion strain and often come with little education and training [36]. These factors regularly contribute to a high level of staff turnover, which was only heightened by the extreme challenges faced during the COVID-19 pandemic, include the increased levels of fear, isolation and infection risk experienced by RCF staff that many respondents reported [36]. The impact of staffing shortages was felt by many divisions in the care and health care field and has been shown to contribute to poor mental health outcomes of current workers due to increased responsibilities and overworked conditions [23]. This indicates an important consideration in planning for future crises. Systems are needed in place to help mitigate the effects of staffing issues. Clear training plans, enhanced recruitment and expedited hiring process of qualified staff can hopefully help to minimize these effects in the future.

Comments centered around mutual support of facility staff and administration and the impact the continued communication and positive interactions with each other helped to lessen the mental load during such a stressful time represents a potential target for meaningful intervention in these facilities. RCFs may benefit from enhanced guidance and training aimed at enhancing the feelings of support and camaraderie among staff during long-lasting stressful emergency situations in care homes.

Several limitations exist in this study. As all respondents were from facilities in California, the application of results to facilities in other states with potentially different RCF organizational structures and licensing requirements may be limited. As confidence levels and other characteristics were self-reported by administrators, this may have led to an overly optimistic representation of actual preparedness or compliance with guidelines within facilities. The survey had a low response of invited participants, resulting in a relatively small sample size, which limited the ability to use other analytic techniques. Finally, the facilities that did not respond to the questionnaire may also have had different experiences and characteristics than those that did respond, which were not able to be assessed in this study. Future research expanding the population surveyed outside of California with a larger sample of RCF administrator/staff participants can help to add meaningful data to the pool of knowledge on this understudied population.

## Conclusion

A large share of older adults are served by small RCFs. Understanding their current and future needs for staff training and resources for information used in a health-related crisis can provide valuable insight for future emergencies. Targeting training efforts to appropriate individuals and ensuring relevant information is included in the resources being used by facility administrators and staff can help to increase efficiency of dissemination of necessary guidelines and information, as well as equip these facilities with the best preparedness tools possible.

Respondents’ comments on staffing and morale challenges during the pandemic echo many similar challenges throughout the care industry during the pandemic. Increased efforts on recruitment and retention of qualified staff, particularly during stressful times is needed and future research can help to identify what can be done to best prepare care facilities, such as RCFs, for future emergencies.

## Data Availability

The dataset presented in this study is not publicly available due to privacy restrictions in accordance with the approval of the California State University, Northridge IRB. The data may be available on request from the corresponding author, pending review and consideration from the university’s IRB.

## Abbreviations

CA: California
CALA: California Assisted Living Association
CalHHS: California Health and Human Services
CCL: Community Care Licensing
CDC: Centers for Disease Control and Prevention
CDPH: California Department of Public Health
COVID-19 or COVID: Coronavirus disease 2019
DSS: Department of Social Services
IRB: Institutional Review Board
PPE: Personal protective equipment
RCF(s): Residential care facilities for older adults, designated in California as residential care facilities for the elderly (RCFEs)
RCFE(s): Residential care facilities for the elderly (the designation for this type of facility in California)
